# Effects of Pharmacotherapy on Combat-Related PTSD, Anxiety, and Depression: A Systematic Review and Meta-Regression Analysis

**DOI:** 10.1371/journal.pone.0126529

**Published:** 2015-05-28

**Authors:** Timothy W. Puetz, Shawn D. Youngstedt, Matthew P. Herring

**Affiliations:** 1 Office of the Director, Clinical Center, National Institutes of Health, Bethesda, Maryland, United States of America; 2 College of Nursing and Health Innovation, Arizona State University, Phoenix, Arizona, United States of America; 3 School of Nutrition and Health Promotion, Arizona State University, Phoenix, Arizona, United States of America; 4 Phoenix VA Health Care System, Phoenix, Arizona, United States of America; 5 Department of Physical Education and Sport Sciences, University of Limerick, Limerick, Ireland; Chiba University Center for Forensic Mental Health, JAPAN

## Abstract

The efficacy of pharmacotherapy for PTSD, anxiety, and depression among combat veterans is not well-established.

**Objectives:**

To estimate the effect of pharmacotherapy on PTSD, anxiety, and depression among combat veterans; to determine whether the effects varied according to patient and intervention characteristics; and to examine differential effects of pharmacotherapy on outcomes.

**Materials and Methods:**

Google Scholar, PILOTS, PsycINFO, PubMed, and Web of Science databases were searched through November 2014. Searches resulted in eighteen double-blind, placebo controlled trials of 773 combat veterans diagnosed with PTSD and included only validated pre- and post-intervention PTSD and anxiety or depression measures. Authors extracted data on effect sizes, moderators, and study quality. Hedges’ *d* effect sizes were computed and random effects models estimated sampling error and population variance. The Johnson-Neyman procedure identified the critical points in significant interactions to define regions of significance.

**Results:**

Pharmacotherapy significantly reduced (Δ, 95%CI) PTSD (0.38, 0.23-0.52), anxiety (0.42, 0.30-0.54), and depressive symptoms (0.52, 0.35-0.70). The effects of SSRIs and tricyclic antidepressants on PTSD were greater than other medications independent of treatment duration. The effect of SSRIs and tricyclic antidepressants were greater than other medications up to 5.2 and 13.6 weeks for anxiety and depression, respectively. The magnitude of the effect of pharmacotherapy on concurrently-measured PTSD, anxiety, and depression did not significantly differ.

**Conclusions:**

Pharmacotherapy reduced PTSD, anxiety, and depressive symptoms in combat veterans. The effects of SSRIs and tricyclic antidepressants were greater for PTSD and occurred quicker for anxiety and depression than other medications.

## Introduction

Posttraumatic stress disorder (PTSD) is a debilitating trauma-related disorder resulting from exposure to a traumatic event or events [1]. PTSD is a pervasive problem among military personnel who have experienced combat [2]. The lifetime prevalence of combat-related PTSD in US combat veterans ranges from approximately 6% to 31% [3]. With over 21.2 million military veterans in the US population, approximately 1.3 to 6.6 million veterans will experience PTSD during their lifetime [4].

Since September 11, 2001, over 2.4 million American service members have served in Iraq or Afghanistan with over 1 million service members deployed twice or more to war zones [5]. Consequently, the Veterans Health Administration and military healthcare systems have seen dramatic increases in cases of combat-related PTSD and depressive and anxiety disorders. Over 54% of the approximately 934,000 OEF/OIF/OND veterans utilizing Veterans Health Administration facilities since 2001 have received diagnosis for a mental health disorder. PTSD (29.4%), depressive disorders (23.2%), and anxiety disorders (20.7%) were the most frequent diagnoses [6].

The 2010 National Defense Authorization Act requested that the Institute of Medicine (IOM) examine the effectiveness of the growing number of PTSD programs and services available to service members and veterans in DoD and VA, respectively. The IOM committee’s report [7] indicated that, although there is a wealth of information on PTSD, there are also substantial gaps in our knowledge of how best to manage PTSD in service members and veterans diagnosed with PTSD [7].

Pharmacotherapy is a common method of treating combat-related PTSD [8]. Several pharmacological approaches have been investigated in the treatment of PTSD (e.g., antidepressants, adrenoreceptor antagonists, anticonvulsants, atypical antipsychotics, benzodiazepines), but the efficacy of pharmacotherapy for PTSD has not been well-established [9]. The aforementioned IOM committee report specifically identified several gaps in PTSD-treatment research in combat-veterans relative to pharmacotherapy to include: (i) further examination of pharmacotherapy for PTSD comorbid with other disorders, and, (ii) concern that although polypharmacy may result in improvement in PTSD symptoms, it may also result in more side effects and contribute to noncompliance to treatment [7]. These issues may be related to the high comorbidity of PTSD with symptoms of other psychological disorders like depression and anxiety or the treatment of specific symptoms (e.g., insomnia, flashbacks) rather than diagnosed psychological disorders [8]. Thus, there is a need to identify which drug classes best manage PTSD symptoms in conjunction with other comorbid psychological symptoms among veterans. These issues are both examined in the current review.

Selective serotonin re-uptake inhibitors (SSRIs) have shown efficacy as a first-line pharmacotherapy, but less than 60% of patients respond to treatment [10]. Other pharmacotherapies have shown similar efficacy to SSRIs, but are less well tolerated and therefore have not become first line therapies [9]. Although the efficacy of different classes of drugs remains uncertain, treating co-occurring disorders and symptoms, such as depression and anxiety, is essential in maximizing treatment outcomes in combat-related PTSD [8]. For example, the National Vietnam Veterans Readjustment Study indicated that 98.9% of veterans with PTSD met criteria for a lifetime comorbid psychiatric diagnosis [11]. The high comorbidity of anxiety and depressive symptoms likely exacerbates the chronic, debilitating effects of PTSD and the resistance to treatment. Thus, there also is a need to identify whether different pharmacological approaches differentially affect PTSD and other comorbid psychological conditions like anxiety and depression.

Although the majority of empirical research has focused on SSRIs, prior reviews have supported the efficacy of numerous short- and long-term pharmacotherapies for PTSD [12, 13], including PTSD diagnoses with comorbid anxiety and depressive symptoms [14]. A prior systematic review which focused on combat-related PTSD supported the efficacy of pharmacotherapy for PTSD among combat veterans [14], but did not focus on the best available evidence (e.g., randomized controlled trials (RCTs) of pharmacotherapy) and was limited by the use of inadequate statistical models. Moreover, no systematic review has examined the potential differential effects of pharmacotherapy across concurrently-measured PTSD, anxiety, and depressive symptoms among combat veterans with PTSD.

Thus, the primary aims of this systematic review were: (1) to estimate the effect size for pharmacotherapy on combat-related PTSD, anxiety, and depressive symptom severity among combat veterans; (2) to determine whether the effects varied according to patient characteristics and modifiable features of pharmacotherapy; and, (3) to examine potential differential effects of pharmacotherapy on concurrently-measured PTSD, anxiety, and depressive symptoms.

## Materials and Methods

This systematic review and meta-analysis was conducted in accordance with the Preferred Reporting Items for Systematic Reviews and Meta-Analyses (PRISMA) statement guidelines [15]. As detailed in the authors’ previously published systematic reviews, standard methods were used for data extraction and quality assessment [16–19], data synthesis and analysis [16–19], meta-regression analysis [16–19], and differential effects analysis [18].

### Data Sources and Searches

Electronic searches of databases were conducted via Google Scholar, PILOTS, PsycINFO, PubMed, and Web of Science from database inception to November 2014 using the search strategy: (Posttraumatic Stress Disorder or PTSD) and (pharmacotherapy or pharmacological treatment) and (combat or combat veteran or military or military personnel or war or war veteran or veteran) and (anxiety or depression). Searches were restricted to randomized controlled trials. Reference lists from retrieved articles were manually searched.

### Study Selection

**Fig 1:**
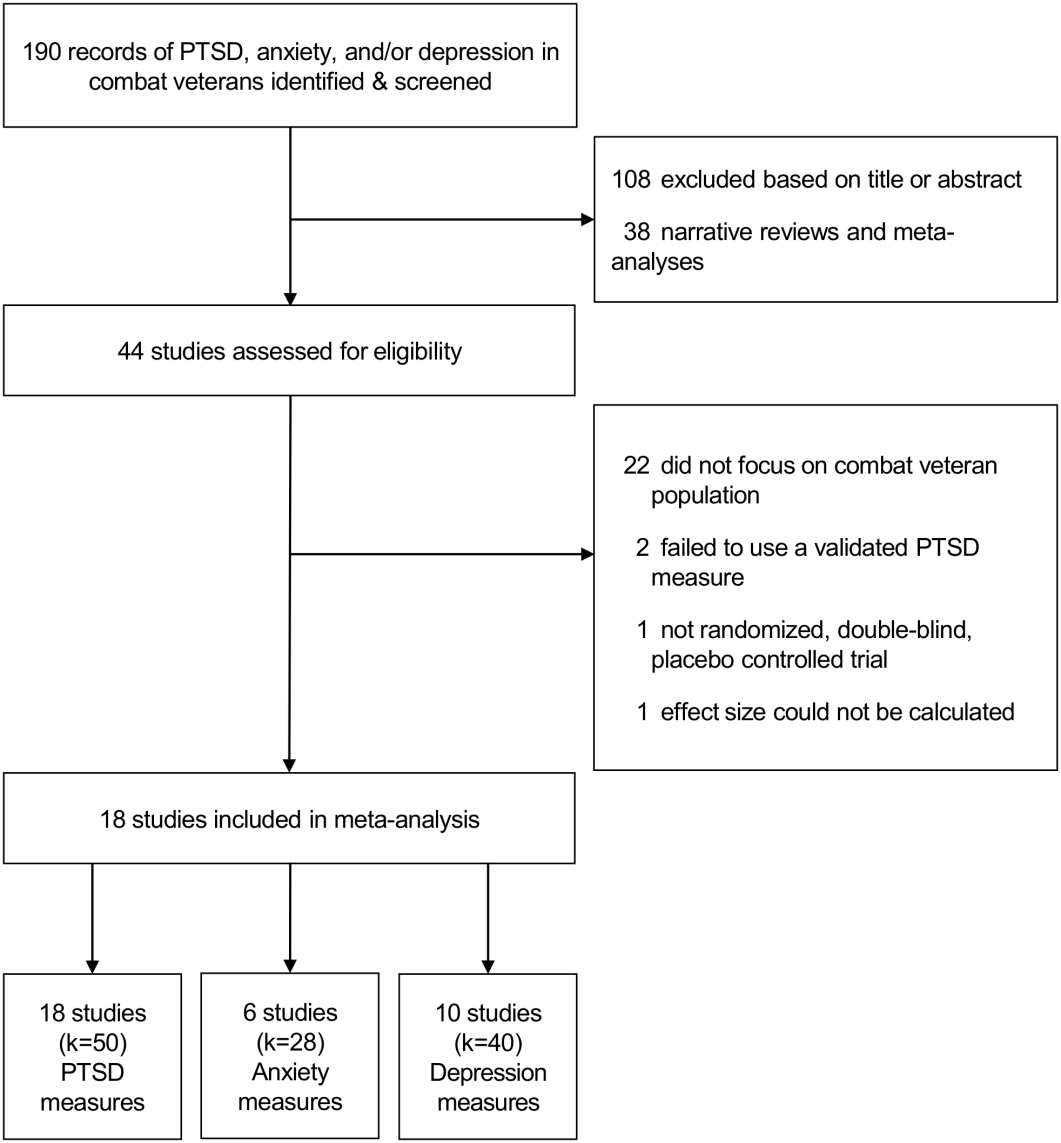
Flow Chart of Study Selection.

Inclusion criteria were: (1) a sample that included only combat veterans diagnosed with PTSD, (2) randomized, double-blind allocation to either pharmacotherapy or placebo condition, and (3) a PTSD symptom severity outcome measured at baseline and during and/or post-intervention. Exclusion criteria were: (1) use of nonrandomized, uncontrolled, or open trial designs; (2) failure to include or specify the inclusion of combat veterans with PTSD; (3) lack of data necessary for the calculation of effect size for PTSD; or (4) failure to use a validated PTSD outcome measure [20]. Fig 1 presents a flowchart of study selection.

### Data Extraction and Quality Assessment

Data were independently extracted by the authors and discrepancies were resolved by consensus judgment. Effect sizes were calculated by subtracting the mean change in the comparison condition from the mean change in the treatment condition and dividing the difference by the pooled standard deviation of baseline scores [21]. Effect sizes were adjusted for small sample size bias and calculated so that decreases in PTSD, anxiety, and depression resulted in positive effect sizes [21]. When a standard deviation was not reported (*k* = 1) [22] it was estimated [23] from the largest study of combat-related PTSD using the same PTSD symptom severity measure [24]. Two-way (Effects x Raters) intraclass correlation coefficients (ICC) for absolute agreement were calculated to examine inter-rater reliability for symptom effect sizes and moderators. The initial ICCs, based on 10 effects, were ≥0.90.

Authors independently assessed study quality using a widely recognized method that addressed randomization, sample selection, quality of outcome measures, and statistical analysis [25]. Quality scores were reported for each study for descriptive purposes, but were not used as weights or moderators in the analysis because of the potential disparity in results that depends on the specific quality scale employed [26].

### Data Synthesis and Analysis

Separate statistical analyses were performed for effects of pharmacotherapy on PTSD, anxiety, and depressive symptom severity. Meta-regression was used as the primary analysis of moderator effects in each of these models in order to reduce the probability of type I error by computing simultaneous estimates of independent effects by multiple moderator variables on the variation in effect size across trials.

An SPSS macro (i.e., *MeanES*; SPSS version 22.0, SPSS Inc., Chicago, IL) was used to calculate the aggregated mean effect size delta (Δ), associated 95% confidence interval, and the sampling error variance according to a random effects model [27]. Random effects models were used to account for between-studies heterogeneity associated with both study-level sampling error and population variance [27]. Each effect was weighted by the inverse of its variance and re-estimated after the random effects variance component was added [21]. Heterogeneity and consistency were evaluated with the Q statistic and the I^2^ statistic, respectively [28]. Heterogeneity also was examined relative to observed variance and was indicated if the sampling error accounted for less than 75% of the observed variance [21]. Publication bias (i.e., smaller studies showing larger effects) was addressed by inspection of a funnel plot [29] and quantified with rank correlation and regression methods [29, 30].

### Primary Moderators

Three primary moderators were selected based on logical, theoretical, or empirical relations to PTSD, anxiety, depression, and/or pharmacotherapy: type of pharmacotherapy, treatment duration, and pharmacotherapy x duration interaction. These variables were tested in each model that met criteria for heterogeneity of effects. Definitions of these variables can be found in S1 Table.

### Primary Moderator Analysis

An SPSS macro (*MetaReg*; SPSS version 22.0, SPSS Inc., Chicago, IL) was used to conduct separate moderator analyses for PTSD, anxiety, and depression symptom severity models [27]. For each model, primary moderator variables were included in a random-effects multiple linear regression analysis with maximum-likelihood estimation [21, 27] adjusted both for non-independence of multiple effects contributed by single studies [31] and for age because of its univariate association with outcomes. Tests of the regression model *(Q*_*R*_ *)* and its residual error *(Q*_*E*_ *)* are reported for each model. Significant categorical moderators in the regression analyses were decomposed using a random effects model to compute mean effect sizes and 95% confidence intervals [27]. The Johnson-Neyman procedure was conducted to identify the critical point in significant interactions of categorical and continuous variables in order to define regions of significance [32, 33].

### Secondary Moderators and Analysis

Secondary moderators were selected for descriptive, univariate analyses for PTSD, anxiety, and depressive symptom severity models. These variables were grouped into patient characteristics (i.e., age, sex, combat sample, baseline symptom score), intervention characteristics (i.e., pharmacotherapy type, program duration, concomitant medication), and study design characteristics (i.e., adherence, time period, outcome measure). Definitions for these variables can be found in S1 Table. Random effects models were used to calculate mean effect sizes (Δ) and 95% confidence intervals for continuous and categorical variables [27].

### Direct Comparison of Concurrent Effects on PTSD, Anxiety, and Depression

Pharmacotherapy investigations that concurrently measured PTSD, anxiety, and depressive symptom severity were used to directly compare the magnitude of the effects among the three mental health outcomes in combat veterans with PTSD. PTSD, anxiety, and depression effect sizes were then dummy coded. Using a SPSS macro (*MetaF*; SPSS version 22.0, SPSS Inc., Chicago, IL), mean effect sizes (Δ) and 95% confidence intervals were computed and the significance of coded effect size variables was tested [27]. Differences among the effects for PTSD, anxiety, and depressive symptoms were determined using the *Q*_*B*_ statistic [21]. For significant tests, pairwise contrasts were tested at p<0.05. Random effects models were used for all analyses [27].

## Results

Eighteen trials of 773 combat veterans were included in the meta-analysis and are presented in S1 References. Characteristics of included trials and study quality assessment are present in Table 1. An annotated table of descriptors of the unweighted Hedge’s *d* effects is presented in Table 2. Funnel plots for all analysis models were inspected and found to be roughly symmetrical (S1, S2, and S3 Figs). The Begg’s rank correlation and Egger’s regression analyses were not statistically significant for any of the models suggesting absence of publication bias (S2 Table).

**Table 1:** Characteristics of Included Trials.

	PTSD		Anxiety		Depression	
**Study Characteristics:**						
**Studies** (n)	18		6		10	
**Effects** (k)	50		28		40	
**Total Sample** (N)	773		365		550	
**Patient Characteristics:**						
**Age** (mean, [SD])	47.3 (7.8)		47.7 (8.7)		47.1 (8.8)	
**Male** (%)	98.3		98.6		98.2	
**Time Since PTSD Diagnosis** (years [SD])	19.0 (9.9)		16.4 (9.2)		19.7 (9.8)	
**Number of Studies Reporting Time Since PTSD Diagnosis** (n [%])	5 (27.8)		2 (33.3)		3 (30.0)	
**Baseline Score** (T-Score [SD])	61.5 (11.1)		63.8 (7.4)		72.1 (11.8)	
**Intervention Characteristics:**						
**Type of Medication** (%)						
Anticonvulsant	22.0		35.7		25.0	
Antipsychotic	6.0		3.6		2.5	
Novel Class	2.0		0.0		0.0	
SSRI	24.0		28.6		20.0	
Tricyclic	28.0		25.0		35.0	
Other	18.0		7.1		17.5	
**Treatment Duration** (weeks [SD])	9.8 (5.3)		10.0 (6.6)		9.8 (5.9)	
**Treatment Adherence** (% [SD])	80.1 (13.9)		83.5 (10.5)		78.6 (13.9)	
**Number of Studies Reporting Treatment Adherence** (n [%])	12 (66.7)		5 (83.3)		8 (80.0)	
**Most Frequently Used Outcome Measures** (k [%])	**CAPS:**	16 (32.0)	**HAM-A:**	26 (92.9)	**MADRS:**	18 (42.5)
	**CGI-S:**	9 (18.0)	**BAI:**	2 (7.1)	**HAM-D:**	17 (45.0)
**Study Quality:**						
**Study Quality** (mean rating [SD])	11.6 (0.9)		12.4 (0.9)		12.1 (0.9)	

**Abbreviations:** SSRI, Selective Serotonin Reuptake Inhibitor; CAPS, Clinician Administered PTSD Scale; CGI-S, Clinical Global Impression-Severity; HAM-A, Hamilton Anxiety Rating Scale; BAI, Beck Anxiety Inventory; MADRS, Montgomery–Åsberg Depression Rating Scale; HAM-D, Hamilton Depression Rating Scale.

**Table 2:** Annotated Descriptors of Unweighted Hedges’ *d* Effect Sizes.

Source	Total N	Age (years)	Sample	Drug Class	Drug Name	Duration (weeks)	Adherence (%)	PTSD Scale	Hedges’ d (95%CI)
Bartzokis et al., 2004	48	51.6	US Vietnam Veterans	Antipsychotic	Risperidone	16	NR	CAPS	0.67 (0.09 to 1.26)
Batki et al., 2014	30	50	US Mixed Conflict Veterans	Anticonvulsant	Topiramate	12	90	PCL-M	0.49 (-0.24 to 1.21)
Davidson et al., 1990	40	53.6	US Mixed Conflict Veterans	TCA	Amitriptyline	8	71	CGI-S	0.98 (0.33 to 1.64)
Davidson et al., 1990	40	53.6	US Mixed Conflict Veterans	TCA	Amitriptyline	8	71	SIP	0.07 (-0.55 to 0.70)
Davidson et al., 1990	40	53.6	US Mixed Conflict Veterans	TCA	Amitriptyline	8	71	IES	0.41 (-0.22to 1.04)
Davidson et al., 1990	33	53.6	US Mixed Conflict Veterans	TCA	Amitriptyline	8	71	CGI-S	0.81 (0.10 to 1.52)
Davidson et al., 1990	33	53.6	US Mixed Conflict Veterans	TCA	Amitriptyline	8	71	SIP	0.26 (-0.43 to 0.94)
Davidson et al., 1990	33	53.6	US Mixed Conflict Veterans	TCA	Amitriptyline	8	71	IES	0.53 (-0.16 to 1.23)
Davis et al., 2004	41	53.8	US Vietnam Veterans	TCA	Nefazodone	4	56	CAPS	0.90 (0.23 to 1.56)
Davis et al., 2004	41	53.8	US Vietnam Veterans	TCA	Nefazodone	8	56	CAPS	0.99 (0.32 to 1.66)
Davis et al., 2004	41	53.8	US Vietnam Veterans	TCA	Nefazodone	12	56	CAPS	0.29 (-0.35 to 0.93)
Davis et al., 2004	41	53.8	US Vietnam Veterans	TCA	Nefazodone	4	56	PCL-M	0.29 (-0.35 to 0.92)
Davis et al., 2004	41	53.8	US Vietnam Veterans	TCA	Nefazodone	8	56	PCL-M	0.26 (-0.38 to 0.90)
Davis et al., 2004	41	53.8	US Vietnam Veterans	TCA	Nefazodone	12	56	PCL-M	0.38 (-0.26 to 1.02)
Davis et al., 2008	82	55.2	US Mixed Conflict Veterans	Anticonvulsant	Divalproex	4	83	CAPS	-0.05 (-0.48 to 0.38)
Davis et al., 2008	82	55.2	US Mixed Conflict Veterans	Anticonvulsant	Divalproex	8	83	CAPS	-0.08 (-0.51 to 0.35)
Davis et al., 2008	82	55.2	US Mixed Conflict Veterans	Anticonvulsant	Divalproex	2	83	TOP-8	-0.04 (-0.47 to 0.39)
Davis et al., 2008	82	55.2	US Mixed Conflict Veterans	Anticonvulsant	Divalproex	4	83	TOP-8	-0.02 (-0.45 to 0.41)
Davis et al., 2008	82	55.2	US Mixed Conflict Veterans	Anticonvulsant	Divalproex	6	83	TOP-8	-0.04 (-0.47 to 0.39)
Davis et al., 2008	82	55.2	US Mixed Conflict Veterans	Anticonvulsant	Divalproex	8	83	TOP-8	0.02 (-0.41 to 0.45)
Davis et al., 2008	82	55.2	US Mixed Conflict Veterans	Anticonvulsant	Divalproex	2	83	CGI-S	-0.10 (-0.54 to 0.33)
Davis et al., 2008	82	55.2	US Mixed Conflict Veterans	Anticonvulsant	Divalproex	4	83	CGI-S	-0.31 (-0.75 to 0.12)
Davis et al., 2008	82	55.2	US Mixed Conflict Veterans	Anticonvulsant	Divalproex	6	83	CGI-S	0.00 (-0.43 to 0.43)
Davis et al., 2008	82	55.2	US Mixed Conflict Veterans	Anticonvulsant	Divalproex	8	83	CGI-S	-0.21 (-0.64 to 0.23)
Frank et al., 1988	23	38	US Vietnam Veterans	TCA	Imipramine	8	NR	IES	0.80 (-0.05 to 1.65)
Frank et al., 1988	22	38	US Vietnam Veterans	MAOI	Phenelzine	8	NR	IES	1.68 (0.71 to 2.65)
Germain et al., 2012	33	40.9	US Mixed Conflict Veterans	Antihypertensive	Prazosin	8	85	PCL-M	0.66 (-0.05 to 1.36)
Germain et al., 2012	33	40.9	US Mixed Conflict Veterans	Antihypertensive	Prazosin	8	70	PCL-M	0.77 (0.06 to 1.47)
Hamner et al., 2003	37	52	US Vietnam Veterans	Antipsychotic	Risperidone	5	NR	CAPS	-0.06 (-0.70 to 0.59)
Hertzberg et al., 2000	12	46	US Vietnam Veterans	SSRI	Fluoxetine	12	92	DTS	-0.27 (-1.40 to 0.87)
Hertzberg et al., 2000	12	46	US Vietnam Veterans	SSRI	Fluoxetine	12	92	SIP	0.00 (-1.13 to 1.13)
Martenyi et al., 2006	144	36.2	European Veterans	SSRI	Fluoxetine	12	97	TOP-8	0.95 (0.55 to 1.35)
Martenyi et al., 2006	144	36.2	European Veterans	SSRI	Fluoxetine	12	97	CAPS	0.97 (0.57 to 1.37)
Martenyi et al., 2006	144	36.2	European Veterans	SSRI	Fluoxetine	12	97	DTS	0.60 (0.21 to 1.00)
Martenyi et al., 2006	144	36.2	European Veterans	SSRI	Fluoxetine	12	97	CGI-S	1.36 (0.95 to 1.78)
Martenyi et al., 2006	144	36.2	European Veterans	SSRI	Fluoxetine	24	97	TOP-8	0.63 (0.12 to 1.13)
Martenyi et al., 2006	144	36.2	European Veterans	SSRI	Fluoxetine	24	97	CAPS	0.42 (-0.09to 0.92)
Martenyi et al., 2006	144	36.2	European Veterans	SSRI	Fluoxetine	24	97	DTS	0.31 (-0.19to 0.81)
Martenyi et al., 2006	144	36.2	European Veterans	SSRI	Fluoxetine	24	97	CGI-S	1.63 (1.05 to 2.20)
Monnelly et al., 2003	15	51.2	US Mixed Conflict Veterans	Antipsychotic	Risperidone	6	NR	PCL-M	0.75 (-0.30 to 1.79)
Neylan et al., 2006	54	44	US Mixed Conflict Veterans	Antihypertensive	Guanfacine	8	90	CAPS	-0.08 (-0.61 to 0.45)
Neylan et al., 2006	54	44	US Mixed Conflict Veterans	Antihypertensive	Guanfacine	8	90	IES	-0.30 (-0.84 to 0.23)
Petrakis et al., 2006	43	46.3	US Mixed Conflict Veterans	ADI	Disulfiram	12	NR	CAPS	0.20 (-0.50 to 0.90)
Petrakis et al., 2006	43	46.3	US Mixed Conflict Veterans	Opioid Antagonist	Naltrexone	12	NR	CAPS	-0.06 (-0.76 to 0.64)
Raskind et al., 2003	10	53	US Vietnam Veterans	Antihypertensive	Prazosin	10	100	CAPS	1.29 (-0.07 to 2.65)
Raskind et al., 2013	67	30.4	US Mixed Conflict Veterans	Antihypertensive	Prazosin	15	69	CAPS	0.48 (-0.01 to 0.97)
Reist et al., 1989	18	38.4	US Vietnam Veterans	TCA	Desipramine	4	67	IES	0.10 (-0.83 to 1.02)
Rothbaum et al., 2008	77	42	US Mixed Conflict Veterans	SNRI	Venlafaxine	12	73	CAPS	-0.11 (-0.84 to 0.62)
Zohar et al., 2002	42	39.5	Israeli Veterans	SSRI	Sertaline	10	NR	CAPS	0.40 (-0.21 to 1.02)
Zohar et al., 2002	42	39.5	Israeli Veterans	SSRI	Sertaline	10	NR	CGI-S	0.41 (-0.20 to 1.03)

**Abbreviations:** ADI, Aldehyde Dehydogenase Inhibitor; MAOI, Monoamine Oxidase Inhibitor; SNRI, Seratonin-Norepinephrine Reuptake Inhibitor; SSRI, Selective Serotonin Reuptake Inhibitor; TCA, Tricyclic Antidepressant; NR, Not Reported; CAPS, Clinician Administered PTSD Scale; PCL-M, PTSD Checklist-Military; TOP-8, Treatment Outcome PTSD Scale; CGI-S, Clinical Global Impression-Severity; SIP, Structured Interview for PTSD; IES, Impact of Events Scale; DTS, Davidson Trauma Scale.

### PTSD Symptom Severity

**Fig 2:**
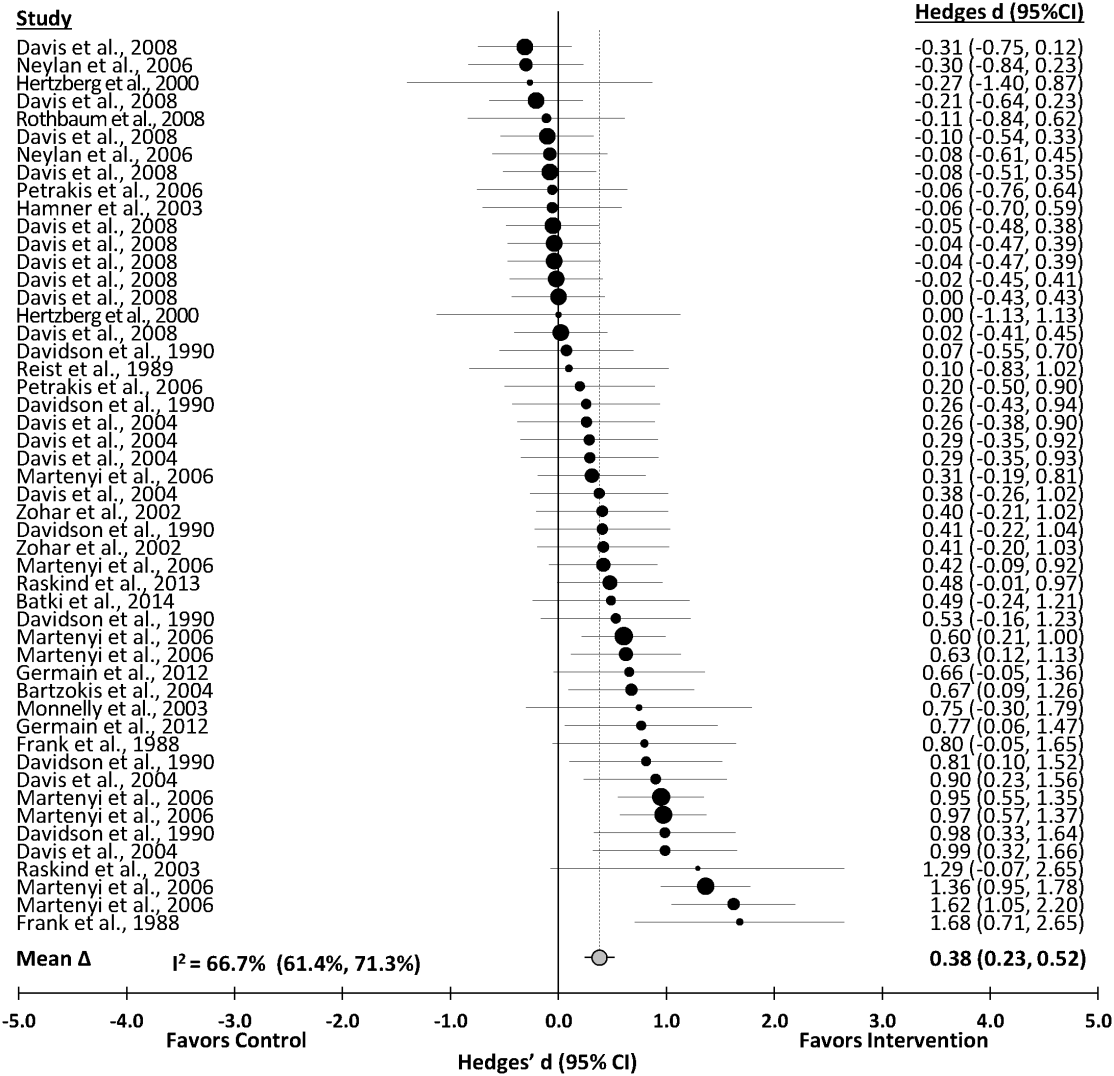
Forest Plot of the Unweighted Distribution of Pharmacotherapy Effects on PTSD.

PTSD symptom severity was significantly reduced after pharmacotherapy interventions (Δ = 0.38 (0.23 to 0.52); *z* = 5.24, p<0.001). The distribution of the effects is presented in Fig 2. The effect was heterogeneous (*Q*_T_(49) = 144.23, p<0.001). Sampling error accounted for 38.2% of the observed variance. The effect was not consistent across studies (I^2^ = 66.7%; 95% CI, 61.4% to 71.3%).

### Moderator analysis

The overall multiple regression model for PTSD was significantly related to effect size (*Q*_*R*_(5) = 58.14; *p*< 0.001, R^2^ = 0.50; *Q*_*E*_(44) = 59.01, *P* = 0.065). Only type of pharmacotherapy (β = 0.77, *z* = 3.15, *P* = 0.002) was independently related to effect size. Significantly larger effects were found in SSRIs and Tricyclic anti-depressants (Δ = 0.63, [95% CI, 0.48, 0.78]) compared to the average of effect of all other drug therapies (Δ = 0.10, [95% CI, -0.05, 0.25]; Q_B_(1) = 23.37, p<0.001).

### Anxiety Symptom Severity

**Fig 3:**
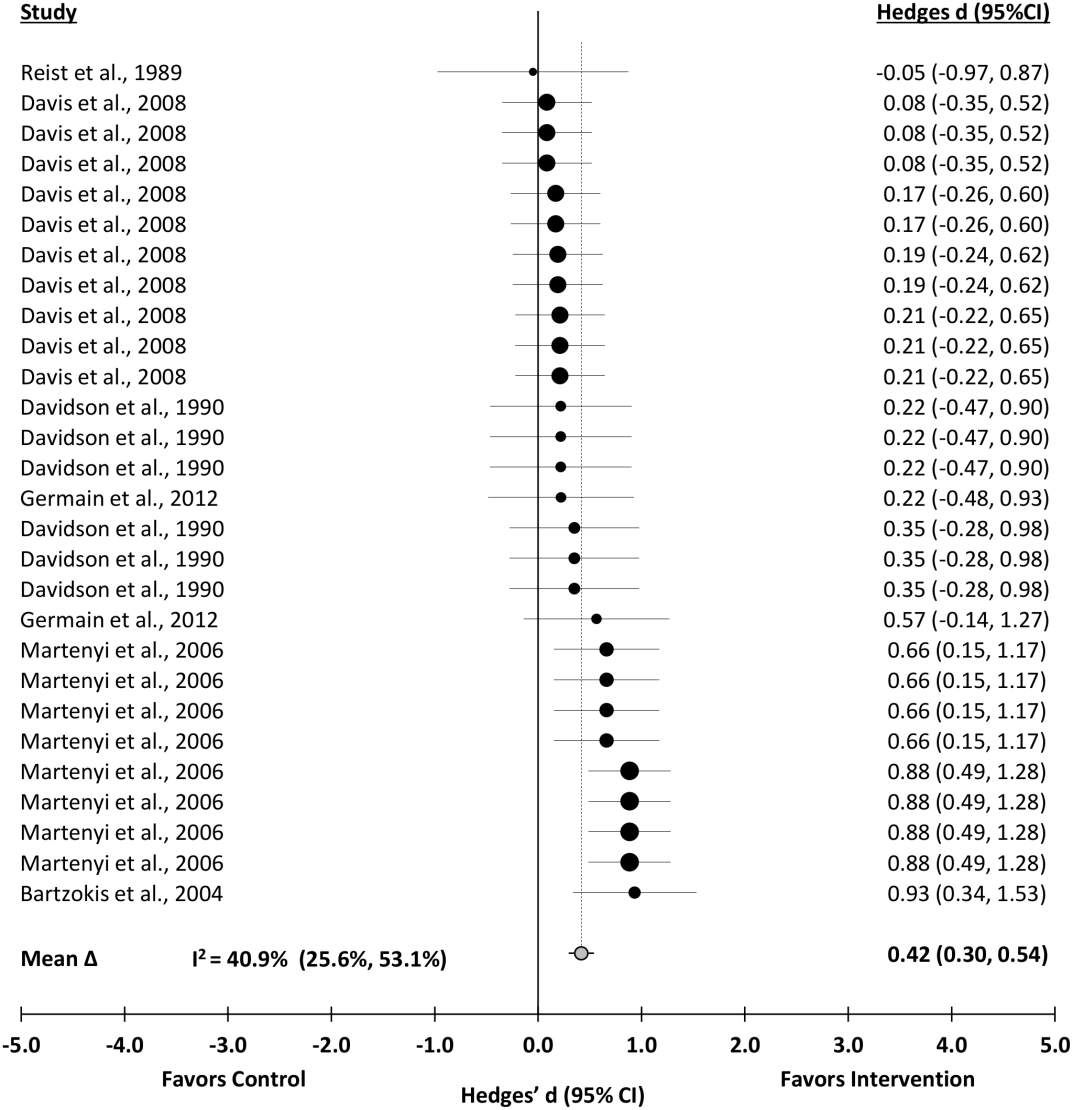
Forest Plot of the Unweighted Distribution of Pharmacotherapy Effects on Anxiety.

Anxiety was significantly reduced after pharmacotherapy interventions (Δ = 0.42 (0.30 to 0.54); *z* = 6.75, p<0.001). A distribution of effects is presented in Fig 3. The effect was heterogeneous (*Q*_T_(27) = 44.01, p = 0.0206). Sampling error accounted for 62.7% of the observed variance. The effect was moderately consistent across studies (I^2^ = 40.9, 95% CI, 25.6% to 53.1%).

#### Moderator analysis

The overall multiple regression model for anxiety was significantly related to effect size (*Q*_*R*_(5) = 36.99; *p*< 0.001, R^2^ = 0.84; *Q*_*E*_(22) = 7.02, *P* = 0.999). The pharmacotherapy x duration interaction (β = 0.06, *z* = 1.94, *P* = 0.050) was independently related to effect size. The Johnson-Neyman procedure yielded critical points for treatment duration at 5.2 (t = -2.07, p = 0.050) and 11.0 weeks (t = 2.07, p = 0.050) when comparing the effects of pharmacotherapy treatment using SSRIs and Tricyclic antidepressants and other drug classes.

### Depressive Symptom Severity

**Fig 4:**
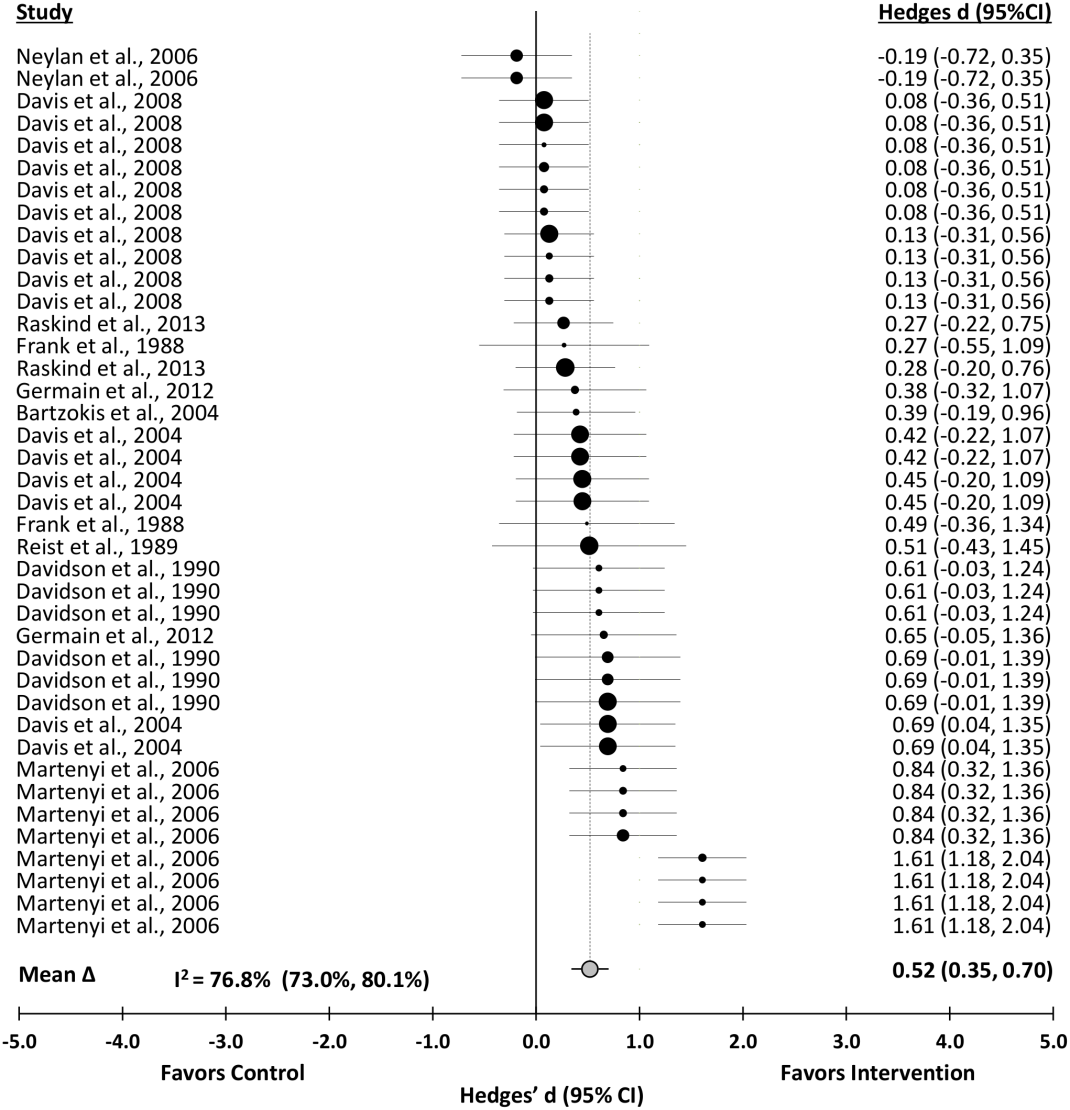
Forest Plot of the Unweighted Distribution of Pharmacotherapy Effects on Depression.

Depression was significantly reduced after pharmacotherapy interventions (Δ = 0.52 (0.35 to 0.70); *z* = 5.81, p< 0.001). A distribution of effects is presented in Fig 4. The effect was heterogeneous (*Q*_T_(38) = 159.58, p<0.001). Sampling error accounted for 42.6% of the observed variance. The effect was not consistent across studies (I^2^ = 76.8%; 95% CI, 73.0% to 80.1%).

#### Moderator analysis

The overall multiple regression model for depression was significantly related to effect size (*Q*_*R*_(5) = 136.57; *p*< 0.001, R^2^ = 0.85; *Q*_*E*_(34) = 23.96, *P* = 0.900). The pharmacotherapy x duration interaction (β = 0.06, *z* = 2.22, *P* = 0.026) was independently related to effect size. The Johnson-Neyman procedure yielded a critical point for treatment duration at 13.6 weeks (t = -2.03, p = 0.050) when comparing the effects of pharmacotherapy treatment using SSRIs and Tricyclic antidepressants and other drug classes.

### Secondary Moderator Analyses

The number of effects (k), mean effect size (Δ), 95% CI, *p* value, and I^2^ for each level of each moderator for PTSD, anxiety, and depression models are presented in S3 Table, S4 Table and S5 Table, respectively.

### Differential Effects Analysis

A total of 28 effects concurrently measured PTSD, anxiety, and depressive symptom severity in combat veterans with PTSD. These effects were derived from 6 studies (N = 365) which had an average sample size of 61 (range = 18 to 144). Direct comparisons indicated no significant difference in the magnitude of the effect of pharmacotherapy on PTSD, anxiety, and depressive symptom severity (*Q*_*B*_ *(2)* = 3.36, p = 0.186). These effects were further decomposed by type of medication for descriptive purposes (S4 Fig).

## Discussion

The cumulative evidence summarized in this review indicates that pharmacotherapy significantly reduces PTSD, anxiety, and depressive symptom severity among combat veterans with PTSD. The magnitude of the overall effects of pharmacotherapy on PTSD (Δ = 0.38), anxiety (Δ = 0.42), and depressive symptoms (Δ = 0.52) were moderate and similar to effects seen in previous reviews of pharmacotherapeutic effects on PTSD, anxiety, and depressive symptoms in non-veteran group [12–14, 34–36]. The reduction in PTSD, anxiety, and depressive symptoms found among combat veterans using pharmacotherapy is equivalent to a number needed to treat [37] of approximately 6 (4.0 to 8.8), 5 (3.8 to 7.0), and 4 (3.0 to 5.8), respectively. Differential analyses showed that pharmacotherapy does not elicit significantly different effects on PTSD, anxiety, and depressive symptoms. These findings support the use of pharmacotherapy as a concurrent treatment for PTSD, anxiety, and depression among combat veterans.

Heterogeneous main effects of pharmacotherapy for PTSD, anxiety, and depression required further examination of the specific treatment characteristics moderating the relationship. The type of medication and the duration of the treatment were especially prominent factors, and are discussed in greater detail in the following sections.

### PTSD Symptom Severity

Reductions in PTSD symptom severity in response to pharmacotherapy among combat veterans were greater for SSRI and Tricyclic antidepressants (Δ = 0.63) compared with other medications (Δ = 0.10) regardless of treatment duration. These findings support the involvement of the serotonergic (5-HT) and noradrenergic (NE) systems in the etiology of PTSD [38]. For example, evidence of the plausible role of the 5-HT system includes associated genetic variation in a polymorphism of the 5-HT transporter and increased 5-HT neurotransmission in key brain areas (e.g., hippocampus, amygdala) following traumatic events [38]. The present findings also are consistent with previous evidence supporting the efficacy of both SSRI and tricyclic antidepressants in treating symptoms of PTSD despite some literature suggesting resistance to these medication classes in combat-related PTSD [10, 39]. However, it is important to examine these findings in the context of the IOM report which acknowledged the concern that, although polypharmacy may result in improvement in PTSD symptoms, it may also increase side effects and contribute to noncompliance to treatment [7]. Indeed, some evidence has suggested that SSRI and Tricyclic antidepressant treatment may not be optimal for individuals with anxiety symptoms [40], reiterating the call within the IOM report for further examination of pharmacotherapy for PTSD comorbid with other psychological symptoms and disorders [7]. The following sections discuss the present findings for comorbid symptoms of depression and anxiety within this context.

### PTSD and Depressive Symptom Severity

Pharmacotherapy significantly improved comorbid depressive symptoms among combat veterans with PTSD. Improvement in depressive symptom severity among combat veterans with PTSD undergoing pharmacotherapy treatment varied according to an interaction between the type of medication and duration of treatment. The effects of SSRI and Tricyclic antidepressants on depressive symptoms were significantly greater than other medications up to a treatment period of approximately 14 weeks, after which there was no longer significant difference between SSRI and Tricyclic antidepressants and other classes of medication. These findings support previous evidence suggesting that PTSD and depression are highly correlated, but independent, responses to trauma, plausibly resulting in differences in treatment response [40–42]. Clinicians may benefit from addressing these differences throughout the course of pharmacotherapy.

PTSD and depression occur frequently following traumatic exposure both concurrently and as separate disorders [40, 42]. While PTSD and comorbid PTSD/depression are often indistinguishable, previous studies support the existence of depression as a separate construct in the acute aftermath of trauma with its own unique characteristics and its own unique course of recovery [40]. PTSD symptoms are strongly predictive of later depression [40, 42]. For example, non-cognitive factors such as hyperarousal have reliably preceded symptoms of depression [42]. Cognitive factors such as intrusive memories also can begin to differentiate comorbid PTSD/depression from depression alone as soon as three months post-trauma. These cognitive factors may act as a mediator between PTSD and depression [40].

Our findings support the symptom-specific time course linking the bidirectional relation between PTSD and depression. SSRIs and tricyclic antidepressants had a greater effect than other drug classes in the management of PTSD symptoms regardless of treatment duration; whereas, the differential therapeutic effects of these medications were most effective for depressive symptoms until about three and a half months into treatment. In addition to alleviating the core symptoms of PTSD, some SSRIs are also effective in treating common comorbidities, such as depression and anxiety [43]. Thus, SSRIs can address depression symptoms directly and also indirectly through non-cognitive factors (i.e., hyperarousal) which may facilitate prevention of future depressive episodes [40, 42]. The slower onset of therapeutic effects found in other drug classes, such as antipsychotics, may be associated with a mechanism of action related to cognitive factors that can mediate the PTSD and depression relationship [42]. This mediated response may be especially important for those patients that do not respond to short-term treatment with SSRI or tricyclic antidepressants.

Although the IOM has questioned the merit of polypharmacy [7], monotherapy with traditional antidepressants may not be sufficient in patients with combat-related PTSD. For example, atypical antipsychotics are an emerging class of drugs that may help alleviate PTSD symptoms along cognitive symptom dimensions [12, 44]. Future studies should investigate new combinations of pharmacotherapy that may provide improvement in both cognitive and non-cognitive PTSD symptoms and aid the prevention of PTSD/depression comorbidity.

### PTSD and Anxiety Symptom Severity

Pharmacotherapy significantly improved comorbid anxiety symptoms among combat veterans with PTSD. Improvement in anxiety symptom severity among combat veterans with PTSD undergoing pharmacotherapy treatment similarly varied according to an interaction between the type of medication and duration of treatment. The effects of SSRI and Tricyclic antidepressants on PTSD symptoms were significantly greater than other medications up to a treatment period of approximately 5 weeks. However, following 11 weeks of treatment the effects of other medication classes were significantly greater than SSRIs and Tricyclic antidepressants.

Nearly 60% of veterans with PTSD report anxiety symptoms, and 20% have reported a panic attack in the previous month [38]. The results reported here support other findings that antidepressant medications, particularly SSRIs, have been effective in the treatment of not only core symptoms of PTSD but also comorbid conditions including panic disorder, social anxiety disorder, and generalized anxiety disorder [43]. These positive effects are likely related to neural circuits and substrates underlying acute and chronic stress responses and to trauma memory encoding and retrieval [9], and underscore the critical need to further examine effects of pharmacotherapy on comorbid symptoms of anxiety among individuals with PTSD [7].

Comorbid PTSD/anxiety also is important to consider during PTSD treatment. For example, higher depressive symptom severity was found among patients with PTSD and comorbid panic disorder compared to patients without the comorbidity [45]. Thus, comorbid anxiety may complicate not only treatment of PTSD, but also depression based-treatment, as it is related to non-cognitive factors such as hyperarousal. Future studies should investigate how to maximize the use of antidepressant agents in the treatment of not only PTSD and depression following trauma, but also in the context of comorbid anxiety disorders.

### Limitations

Limitations in the adequacy of reporting and the methodological rigor of included trials are of note. Several trials did not provide adequate information about features of the intervention, particularly regarding concomitant medication use and adherence/compliance to the prescribed pharmacotherapy, while others did not utilize the most well-validated outcome measures available. Moreover, the limited number of effects derived from studies that examined novel class treatments such as atypical antipsychotics or novel class antidepressants preclude meaningful interpretations of findings for these drug classes and warrant future research. Finally, given both the prevalence of comorbid anxiety and depressive symptoms in PTSD and the present findings which suggest that pharmacotherapy can concurrently attenuate these symptoms among combat veterans with PTSD, future research should prioritize concurrent assessment of related symptoms.

## Conclusions

PTSD is a pervasive problem among combat veterans. Concurrent anxiety and depressive symptoms are frequently reported among veterans with PTSD, and likely exacerbate the chronic, debilitating effects of PTSD and the resistance to treatment. PTSD currently has few proven pharmacotherapies. However, the evidence reviewed here suggests that pharmacotherapy has a positive, but modest, therapeutic effect on PTSD, anxiety, and depressive symptom severity, and it also successfully acts as a concurrent treatment for these symptoms among combat veterans. This is especially evident for SSRI and Tricyclic antidepressants. The therapeutic effects of SSRI and tricyclic antidepressant medications were greater for PTSD and occurred more quickly for anxiety and depression than with other commonly prescribed medications. While the pathophysiology of PTSD implicates many different neurotransmitter and neuroanatomical pathways, the delineation of the abnormalities in these chemical, structural, and neural systems will require time to fully understand. Until that time, the available evidence suggests that SSRIs and Tricyclic antidepressants should be considered a first-line treatment while making an allowance for other emerging classes of medication that may further alleviate symptoms in refractory PTSD relative to cognitive dimensions such as avoidance and intrusive memories.

## Supporting Information

S1 Fig Funnel Plot for PTSD Effects. (TIF)

S2 Fig Funnel Plot for Anxiety Effects. (TIF)

S3 Fig Funnel Plot for Depression Effects. (TIF)

S4 Fig Mean Effect of Pharmacotherapy on PTSD, Anxiety, and Depression. (TIF)

S1 References References of Included Trials. (DOCX)

S1 Table Definitions of Levels of Moderators. (DOCX)

S2 Table Statistical Tests for Publication Bias. (DOCX)

S3 Table Summary of Univariate Moderator Analysis for Pharmacotherapy Effects on PTSD Symptoms. (DOCX)

S4 Table Summary of Univariate Moderator Analysis for Pharmacotherapy Effects on Anxiety Symptoms. (DOCX)

S5 Table Summary of Univariate Moderator Analysis for Pharmacotherapy Effects on Depressive Symptoms. (DOCX)
