# Supplementary figures and images for: Effects of Pharmacotherapy on Combat-Related PTSD, Anxiety, and Depression: A Systematic Review and Meta-Regression Analysis
Source: PLoS One. 2015 May 28;10(5):e0126529. doi: 10.1371/journal.pone.0126529 (PMC4447407; doi:10.1371/journal.pone.0126529)

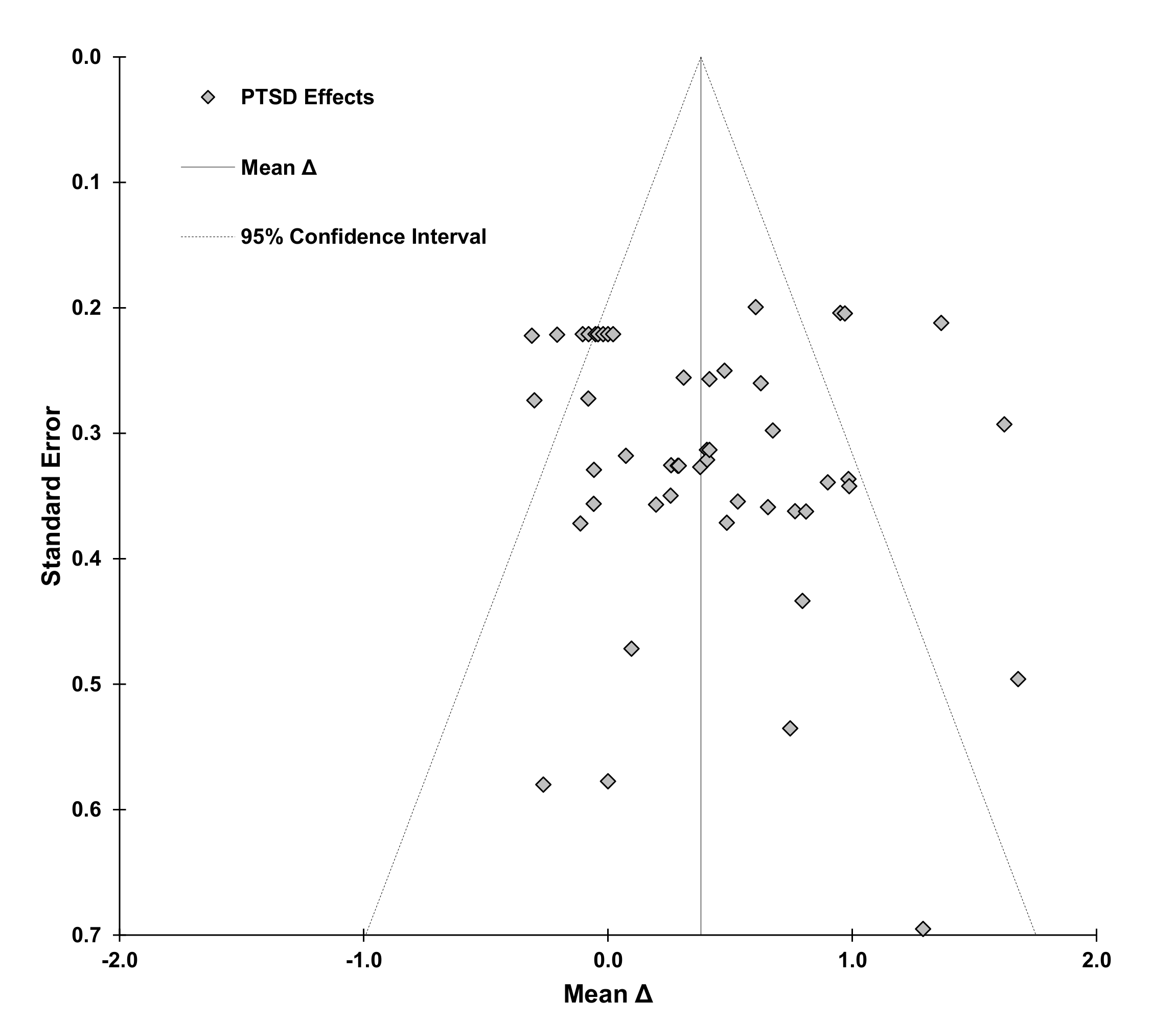

Supplement: S1 Fig — (TIF) [file pone.0126529.s001.tif]

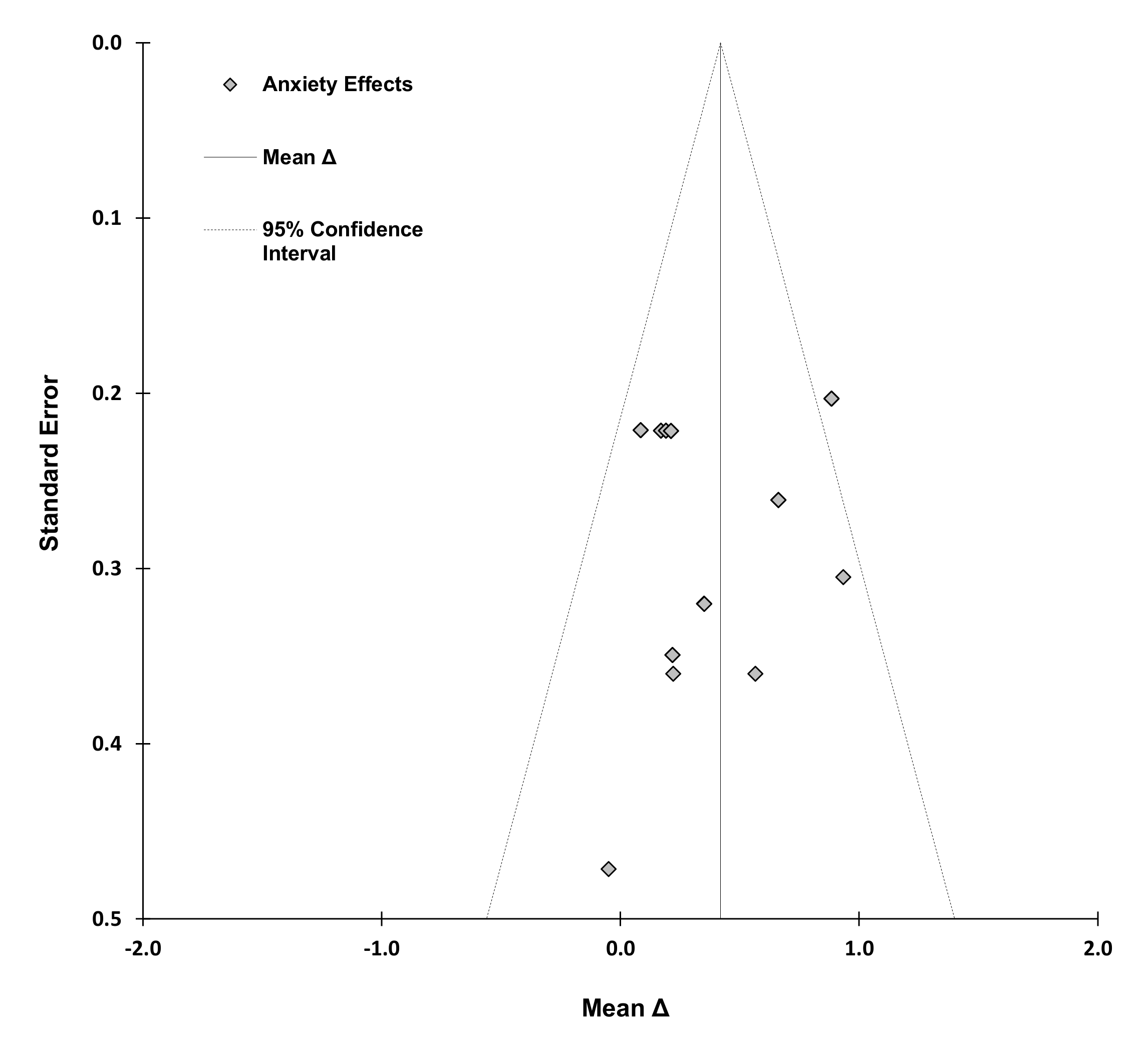

Supplement: S2 Fig — (TIF) [file pone.0126529.s002.tif]

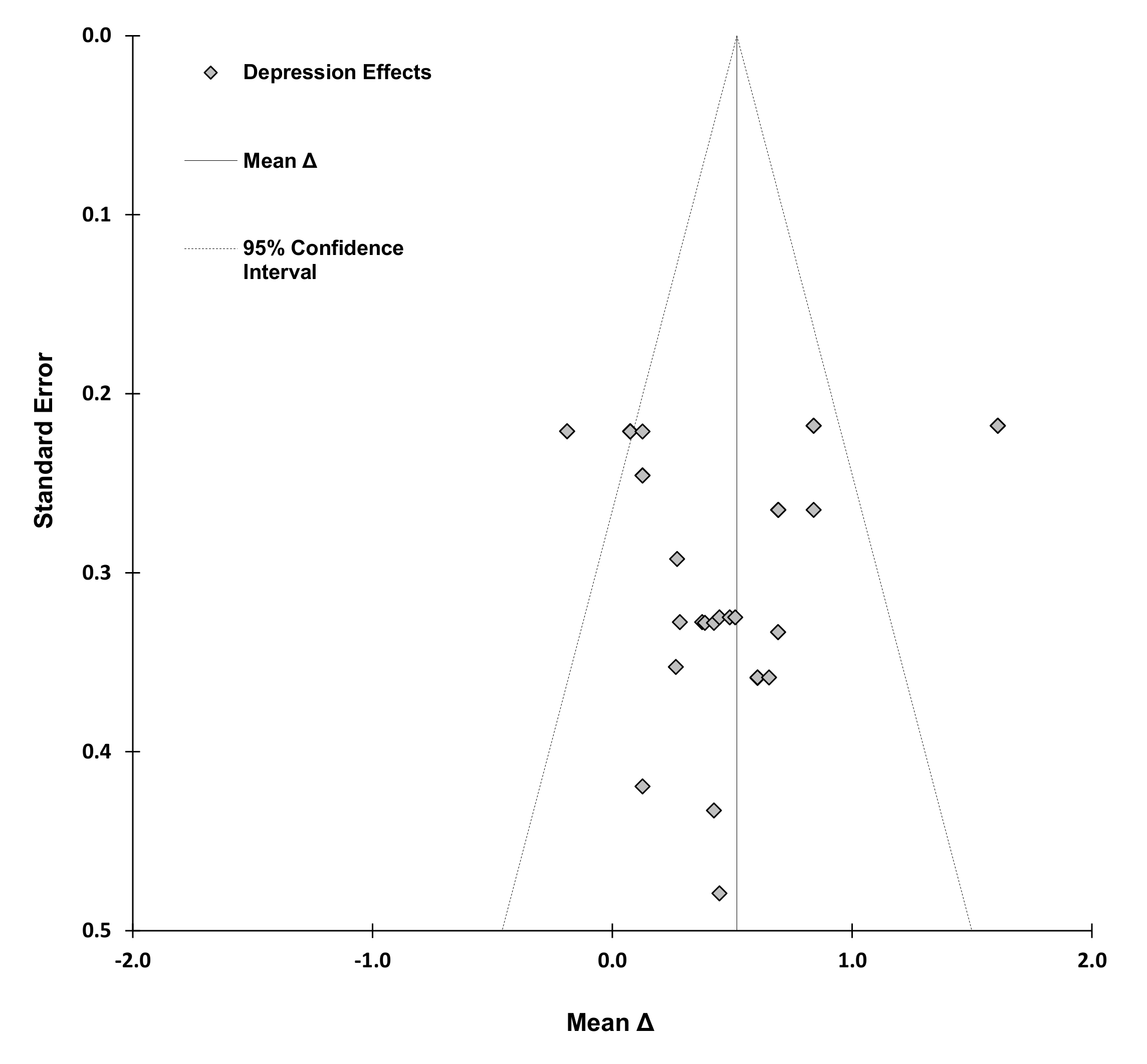

Supplement: S3 Fig — (TIF) [file pone.0126529.s003.tif]

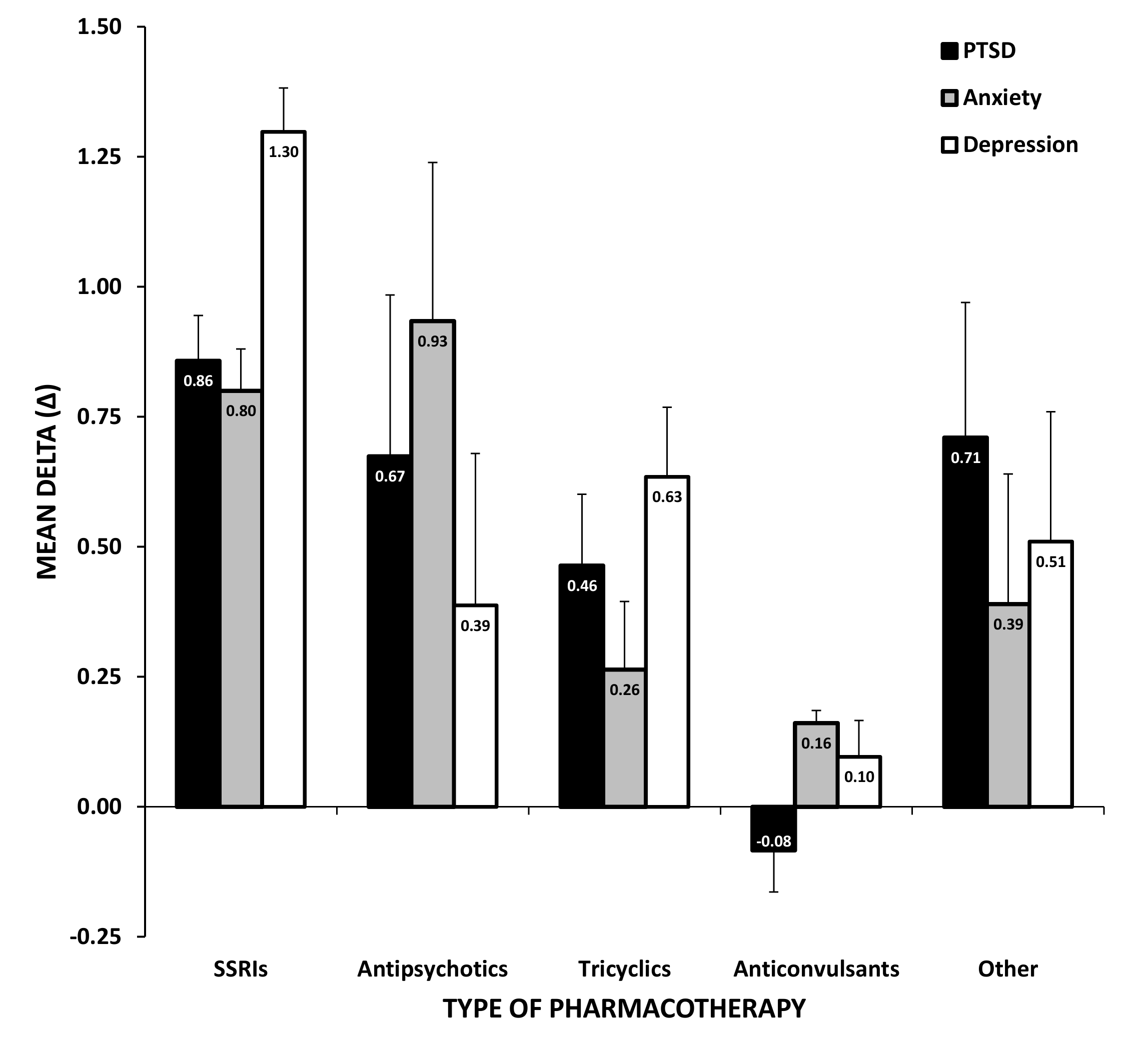

Supplement: S4 Fig — (TIF) [file pone.0126529.s004.tif]
