# Supplementary material for: Effects of Pharmacotherapy on Combat-Related PTSD, Anxiety, and Depression: A Systematic Review and Meta-Regression Analysis
Source: PLoS One. 2015 May 28;10(5):e0126529. doi: 10.1371/journal.pone.0126529 (PMC4447407; doi:10.1371/journal.pone.0126529)
Supplement: S1 References — (DOCX) [file pone.0126529.s005.docx]

**S1 References. References of Included Trials.**

1. Bartzokis G, Lu PH, Turner J, Mintz J, Saunders CS. Adjunctive risperidone in the treatment of chronic combat-related Posttraumatic Stress Disorder. *Biol Psychiatry*. 2004;57(5):474-479.
2. Batki SL, Pennington BL, Neylan TC, et al. Topiramate treatment of alcohol use disorder in veterans with posttraumatic stress disorder: A randomized controlled pilot trial. *Alcohol Clin Exp Res*. 2014; 38(8):2169-77.
3. Davidson J, Kudler H, Smith R, et al. Treatment of Posttraumatic Stress Disorder with amitriptyline and placebo. *Arch Gen Psychiatry*. 1990; 47(3):259-266.
4. Davis LL, Jewell ME, Ambrose S, et al. A placebo-controlled study of nefazodone for the treatment of chronic Posttraumatic Stress Disorder: A preliminary study. *J Clin Psychopharmacol*. 2004; 24(3):291-297.
5. Davis LL, Davidson JRT, Ward LC, Bartolucci A, Bowden CL, Petty F. Divalproex in the treatment of Posttraumatic Stress Disorder. A randomized, double-blind, placebo-controlled trial in a veteran population. *J Clin Psychopharmacol*. 2008; 28(1):84-88.
6. Frank JB, Kosten TR, Giller EL, Dan E. A randomized clinical trial of phenelzine and imipramine for Posttraumatic Stress Disorder. *Am J Psychiatry*. 1988; 145(10):1289-1291.
7. Germain A, Richardson R, Moul DE, et al. Placebo-controlled comparison of prazosin and cognitive-behavioral treatments for sleep disturbances in US Military Veterans. *J Psychosom Res*. 2012; 72(2):89-96.
8. Hamner MB, Faldowski RA, Ulmer HG, Frueh BC, Huber MG, Arana GW. Adjunctive risperidone treatment in post-traumatic stress disorder: A preliminary controlled trial of effects on comorbid symptoms. *Int Clin Psychopharmacol*. 2003; 18(1):1-8.
9. Hertzberg MA, Feldman ME, Beckham JC, Kudler HS, Davidson JRT. Lack of efficacy for fluoxetine in PTSD: A placebo controlled trial in combat veterans. *Ann Clin Psychiatry*. 2000; 12(2):101-105.
10. Martenyi F, Soldatenkova V. Fluoxetine in the acute treatment and relapse prevention of combat-related post-traumatic stress disorder: Analysis of the veteran group of a placebo-controlled, randomized clinical trial. *Eur Neuropsychopharmacol*. 2006; 16(5):340-349.
11. Monnelly DP, Ciraul DA, Knapp C, Keane T. Low-dose risperidone as adjunctive therapy for irritable aggression in Posttraumatic Stress Disorder. *J Clin Psychopharmacol*. 2003; 23(2):193-196.
12. Neylan TC, Lenoci M, Samuelson KW, et al. No improvement of Posttraumatic Stress Disorder symptoms with guanfacine treatment. *Am J Psychiatry*. 2006; 163(12):2186-2188.
13. Petrakis IL, Poling J, Levinson C, et al. Naltrexone and disulfiram in patients with alcohol dependence and comorbid Post-Traumatic Stress Disorder. *Biol Psychiatry*. 2006; 60(7):777-783.
14. Raskind MA, Peskind ER, Kanter ED, et al. Reduction of nightmares and other PTSD symptoms in combat veterans by prazosin: A placebo-controlled study. *Am J Psychiatry*. 2003; 160(2):371-373.
15. Raskind MA, Peterson K, Williams T et al. A trial of prazosin for combat trauma PTSD with nightmares in active-duty soldiers returned from Iraq and Afghanistan. *Am J Psychiatry*. 2013; 170(9):1003-10.
16. Reist C, Kauffmann CD, Haier RJ, et al. A controlled trial of desipramine in 18 men with Posttramatic Stress Disorder. *Am J Psychiatry*. 1989; 146(4):513-516.
17. Rothbaum BO, Davidson JRT, Stein DJ, et al. A pooled analysis of gender and trauma-type effects on responsiveness to treatment of PTSD with venlafaxine extended release or placebo. *J Clin Psychiatry*. 2008; 69(10):1529-1539.
18. Zohar J, Amital D, Miodownik C, et al. Double-blind placebo-controlled pilot study of sertaline in military veterans with Posttraumatic Stress Disorder. *J Clin Psychopharmacol*. 2002; 22(2):190-195.
