# Supplementary material for: Effects of Pharmacotherapy on Combat-Related PTSD, Anxiety, and Depression: A Systematic Review and Meta-Regression Analysis
Source: PLoS One. 2015 May 28;10(5):e0126529. doi: 10.1371/journal.pone.0126529 (PMC4447407; doi:10.1371/journal.pone.0126529)
Supplement: S1 Table — (DOCX) [file pone.0126529.s006.docx]

| **S1 Table. Definitions for Levels of Moderators.** | |
| --- | --- |
| **Effect Moderator** | **Levels** |
|  |  |
| **Patient Characteristics** |  |
|  |  |
| Age | **Continuous variable:** years |
| Sex | **Male:** data from males only |
|  | **Female:** data from females only |
|  | **Mixed:** data from samples that combined males and females |
| Combat Sample | **US. Vietnam Veterans:** data from samples who were United States military combat veterans and served in the Vietnam War |
|  | **Israeli Combat Veterans:** data from samples who were Israeli military combat veterans |
|  | **Mixed:** data from samples that combined combat veterans from different wars |
| Baseline Symptom Score | **Continuous variable:** T-scores |
|  |  |
| **Intervention Characteristics** |  |
|  |  |
| Pharmacotherapy Type | **Anticonvulsant:** the intervention used an anticonvulsant drug |
|  | **Antipsychotic:** the intervention used an antipsychotic drug |
|  | **Novel Class:** the intervention used a novel class drug |
|  | **SSRI:** the intervention used a selective serotonin reuptake inhibitor drug |
|  | **Tricyclic:** the intervention used a tricyclic drug |
|  | **Other:** the intervention used a therapeutic drug not categorized above |
| Program Duration | **Continuous variable:** weeks |
| Concomitant Medication | **Yes:** the patients were taking one or more medications beyond that used in the intervention |
|  | **No:** the patents were not taking any medication beyond that used in the intervention |
|  | **Not Reported:** the study did not report whether or not patients were taking medication beyond that used in the intervention |
|  |  |
| **Study Characteristics** |  |
|  |  |
| Adherence | **Continuous variable:** percentage |
| Time Period | **During Intervention:** the study included measures collected during the intervention |
|  | **Post Intervention:** the study included measures collected immediately following the completion of the intervention |
|  | **Follow Up:** the study included measures collected weeks to months following post-intervention |
| Comparison Type | **Placebo:** the study used a placebo control comparison condition |
|  | **Other:** the study used a comparison not categorized above |

| **S1 Table. Definitions for Levels of Moderators. (Continued)** | |
| --- | --- |
|  |  |
| Outcome Measure | **PTSD** |
|  | **CAPS:** the study used the Clinician Administered PTSD Scale |
|  | **PCL-M:** the study used the PTSD Checklist-Military |
|  | **TOP-8:** the study used the Treatment Outcome PTSD Scale |
|  | **CGI-S:** the study used the Clinical Global Impression-Severity of Illness Scale |
|  | **SIP:** the study used the Structured Interview for PTSD |
|  | **IES:** the study used the Impact of Events Scale |
|  | **DTS:** the study used the Davidson Trauma Scale |
|  | **Anxiety** |
|  | **HAM-A:** the study used the Hamilton Anxiety Scale |
|  | **Depression** |
|  | **HAM-D:** the study used the Hamilton Depression Scale |
|  | **BDI:** the study used the Beck Depression Inventory |
|  | **MADRS:** the study used the Montgomery–Åsberg Depression Rating Scale |
|  | **RDRS:** the study used the Raskin Depression Rating Scale |
|  |  |
