# Supplementary material for: Effects of Pharmacotherapy on Combat-Related PTSD, Anxiety, and Depression: A Systematic Review and Meta-Regression Analysis
Source: PLoS One. 2015 May 28;10(5):e0126529. doi: 10.1371/journal.pone.0126529 (PMC4447407; doi:10.1371/journal.pone.0126529)
Supplement: S2 Table — (DOCX) [file pone.0126529.s007.docx]

| **S2 Table. Statistical Tests for Publication Bias.** | | | |
| --- | --- | --- | --- |
| **BEGG’S RANK CORRELATION** | | | |
| **Model** | **Kendall’s τ** | **DF** | **p-value** |
| PTSD | -0.04 | 49 | 0.185 |
| Anxiety | -0.04- | 27 | 0.774 |
| Depression | -0.01 | 39 | 0.953 |

| **EGGER’S REGRESSION TEST** | | | | |
| --- | --- | --- | --- | --- |
| **Model** | **β** | **t-test** | **DF** | **p-value** |
| PTSD | -0.52 | -0.40 | 48 | 0.694 |
| Anxiety | -1.57 | -0.94 | 26 | 0.355 |
| Depression | -0.50 | -0.23 | 38 | 0.822 |
