# Supplementary material for: Effects of Pharmacotherapy on Combat-Related PTSD, Anxiety, and Depression: A Systematic Review and Meta-Regression Analysis
Source: PLoS One. 2015 May 28;10(5):e0126529. doi: 10.1371/journal.pone.0126529 (PMC4447407; doi:10.1371/journal.pone.0126529)
Supplement: S3 Table — (DOCX) [file pone.0126529.s008.docx]

| **S3 Table. Summary of Univariate Moderator Analysis for Pharmacotherapy Effects on PTSD Symptoms.** | | | | | |
| --- | --- | --- | --- | --- | --- |
| **Effect Moderator** | **Effects (k)** | **Δ or β** | **95% CI** | **p-value** | **I^2^** |
|  |  |  |  |  |  |
| **PTSD Symptom Severity** |  |  |  |  |  |
| **Pharmacotherapy** | 50 | 0.38 | 0.23, 0.52 | 0.0000 | 66.7% |
|  |  |  |  |  |  |
| **Patient Characteristics** |  |  |  |  |  |
| **Age** (years) | 50 | -0.03 | -0.04, -0.02 | 0.0000 | 32.0% |
| **Sex** |  |  |  |  |  |
| Male | 23 | -0.46 | -0.26, 0.68 | 0.0000 | 0.0% |
| Mixed | 24 | -0.37 | -0.19, 0.54 | 0.0000 | 30.7% |
| Not Reported | 3 | -0.17 | -0.70, 0.36 | 0.5275 | 0.0% |
| **Combat Sample** |  |  |  |  |  |
| U.S. Vietnam Veterans | 14 | -0.50 | -0.22, 0.79 | 0.0005 | 0.0% |
| Israeli Combat Veterans | 2 | -0.41 | -0.26, 1.08 | 0.2330 | NA |
| Mixed | 34 | -0.33 | -0.18, 0.49 | 0.0000 | 13.6% |
| **Baseline T-Score** | 50 | -0.01 | -0.01, 0.02 | 0.4187 | 1.0% |
|  |  |  |  |  |  |
| **Intervention Characteristics** |  |  |  |  |  |
| **Pharmacotherapy Type** |  |  |  |  |  |
| Anticonvulsant | 11 | -0.06 | -0.22, 0.11 | 0.4844 | 0.0% |
| Antipsychotic | 3 | -0.40 | -0.04, 0.85 | 0.0744 | 61.7% |
| Novel Class | 1 | -0.11 | -0.91, 0.68 | 0.7801 | NA |
| SSRI | 12 | -0.75 | -0.58, 0.93 | 0.0000 | 57.8% |
| Tricyclic | 14 | -0.50 | -0.30, 0.70 | 0.0000 | 0.0% |
| Other | 9 | -0.32 | -0.08, 0.57 | 0.0101 | 59.9% |
| **Treatment Duration** (weeks) | 50 | -0.04 | -0.01, 0.06 | 0.0016 | 19.7% |
| **Concomitant Medication** |  |  |  |  |  |
| Yes | 12 | 0.30 | -0.07, 0.54 | 0.0106 | 12.5% |
| No | 20 | 0.10 | -0.06, 0.25 | 0.2097 | 0.0% |
| Not Reported | 18 | 0.73 | -0.56, 0.91 | 0.0000 | 39.1% |
|  |  |  |  |  |  |
| **Study Characteristics** |  |  |  |  |  |
| **Adherence** | 41 | 0.37 | -0.74, 1.49 | 0.5145 | 1.0% |
| **Time Period** |  |  |  |  |  |
| During Intervention | 18 | -0.39 | -0.18, 0.60 | 0.0003 | 27.1% |
| Post Intervention | 31 | -0.35 | -0.17, 0.53 | 0.0001 | 0.0% |
| Follow Up | 1 | -0.77 | -0.25, 1.78 | 0.1412 | NA |
| **PTSD Measure** |  |  |  |  |  |
| CAPS | 16 | -0.35 | -0.11, 0.59 | 0.0037 | 0.0% |
| PCL-M | 7 | -0.49 | -0.10, 0.88 | 0.0130 | 0.0% |
| TOP-8 | 6 | -0.25 | -0.10, 0.59 | 0.1612 | 20.3% |
| CGI-S | 9 | -0.46 | -0.17, 0.76 | 0.0023 | 65.9% |
| SIP | 3 | -0.13 | -0.49, 0.74 | 0.6845 | 0.0% |
| IES | 6 | -0.44 | -0.02, 0.87 | 0.0413 | 45.6% |
| DTS | 3 | -0.35 | -0.20, 0.90 | 0.2183 | 14.8% |
