# Supplementary material for: Effects of Pharmacotherapy on Combat-Related PTSD, Anxiety, and Depression: A Systematic Review and Meta-Regression Analysis
Source: PLoS One. 2015 May 28;10(5):e0126529. doi: 10.1371/journal.pone.0126529 (PMC4447407; doi:10.1371/journal.pone.0126529)
Supplement: S4 Table — (DOCX) [file pone.0126529.s009.docx]

| **S4 Table. Summary of Univariate Moderator Analysis for Pharmacotherapy Effects on Anxiety Symptoms.** | | | | | |
| --- | --- | --- | --- | --- | --- |
| **Effect Moderator** | **Effects (k)** | **Δ or β** | **95% CI** | **p-value** | **I^2^** |
|  |  |  |  |  |  |
| **Anxiety Symptom Severity** |  |  |  |  |  |
| **Pharmacotherapy** | 28 | 0.42 | 0.30, 0.54 | 0.0000 | 40.9% |
|  |  |  |  |  |  |
| **Patient Characteristics** |  |  |  |  |  |
| **Age** (years) | 28 | -0.03 | -0.04, -0.02 | 0.0000 | 40.9% |
| **Sex** |  |  |  |  |  |
| Male | 8 | 0.36 | 0.08, 0.63 | 0.0108 | 0.0% |
| Mixed | 20 | 0.43 | 0.30, 0.57 | 0.0000 | 13.3% |
| Not Reported | 0 | NA | NA | NA | NA |
| **Combat Sample** |  |  |  |  |  |
| U.S. Vietnam Veterans | 2 | 0.60 | 0.02, -1.18 | 0.0417 | NA |
| Israeli Combat Veterans | 0 | NA | NA | NA | NA |
| Mixed | 26 | 0.41 | 0.29, -0.53 | 0.0000 | 0.0% |
| **Baseline T-Score** | 28 | -0.02 | -0.03, -0.01 | 0.0320 | 17.1% |
|  |  |  |  |  |  |
| **Intervention Characteristics** |  |  |  |  |  |
| **Pharmacotherapy Type** |  |  |  |  |  |
| Anticonvulsant | 10 | 0.16 | -0.02, 0.30 | 0.0212 | 0.0% |
| Antipsychotic | 1 | 0.93 | -0.34, 1.53 | 0.0022 | NA |
| Novel Class | 0 | NA | NA | NA | NA |
| SSRI | 8 | 0.80 | -0.64, 0.96 | 0.0000 | 0.0% |
| Tricyclic | 7 | 0.26 | -0.01, 0.52 | 0.0439 | 0.0% |
| Other | 2 | 0.39 | -0.11, 0.89 | 0.1235 | NA |
| **Treatment Duration** (weeks) | 28 | 0.03 | -0.02, 0.05 | 0.0000 | 36.1% |
| **Concomitant Medication** |  |  |  |  |  |
| Yes | 3 | 0.61 | -0.23, 0.99 | 0.0017 | 57.3% |
| No | 11 | 0.16 | -0.02, 0.29 | 0.0236 | 0.0% |
| Not Reported | 14 | 0.67 | -0.53, 0.80 | 0.0000 | 3.7% |
|  |  |  |  |  |  |
| **Study Characteristics** |  |  |  |  |  |
| **Adherence** | 27 | 2.58 | 1.55, 3.61 | 0.000 | 39.3% |
| **Time Period** |  |  |  |  |  |
| During Intervention | 14 | 0.42 | -0.25, 0.58 | 0.0000 | 29.1% |
| Post Intervention | 13 | 0.41 | -0.22, 0.60 | 0.0000 | 0.0% |
| Follow Up | 1 | 0.57 | -0.25, 1.38 | 0.1721 | NA |
| **Anxiety Measure** |  |  |  |  |  |
| HAM-A | 26 | 0.42 | -0.29, 0.54 | 0.0000 | 0.2% |
| BAI | 2 | 0.39 | -0.18, 0.96 | 0.1790 | NA |
