# Supplementary material for: Effects of Pharmacotherapy on Combat-Related PTSD, Anxiety, and Depression: A Systematic Review and Meta-Regression Analysis
Source: PLoS One. 2015 May 28;10(5):e0126529. doi: 10.1371/journal.pone.0126529 (PMC4447407; doi:10.1371/journal.pone.0126529)
Supplement: S5 Table — (DOCX) [file pone.0126529.s010.docx]

| **S5 Table. Summary of Univariate Moderator Analysis for Pharmacotherapy Effects on Depressive Symptoms.** | | | | | |
| --- | --- | --- | --- | --- | --- |
| **Effect Moderator** | **Effects (k)** | **Δ or β** | **95% CI** | **p-value** | **I^2^** |
|  |  |  |  |  |  |
| **Depression Symptom Severity** |  |  |  |  |  |
| **Pharmacotherapy** | 40 | 0.52 | 0.35, 0.70 | 0.0000 | 76.8% |
|  |  |  |  |  |  |
| **Patient Characteristics** |  |  |  |  |  |
| **Age** (years) | 40 | -0.03 | -0.04, -0.01 | 0.0001 | 26.2% |
| **Sex** |  |  |  |  |  |
| Male | 16 | -0.54 | -0.28, 0.80 | 0.0000 | 0.0% |
| Mixed | 22 | -0.56 | -0.36, 0.75 | 0.0000 | 41.1% |
| Not Reported | 2 | -0.19 | -0.86, 0.48 | 0.5821 | NA |
| **Combat Sample** |  |  |  |  |  |
| U.S. Vietnam Veterans | 10 | -0.48 | -0.14, -0.82 | 0.0062 | 0.0% |
| Israeli Combat Veterans | 0 | NA | NA | NA | NA |
| Mixed | 30 | -0.52 | -0.35,- 0.70 | 0.0000 | 20.3% |
| **Baseline T-Score** | 40 | -0.01 | -0.02, -0.01 | 0.1009 | 1.6% |
|  |  |  |  |  |  |
| **Intervention Characteristics** |  |  |  |  |  |
| **Pharmacotherapy Type** |  |  |  |  |  |
| Anticonvulsant | 10 | -0.10 | -0.04, 0.23 | 0.1690 | 0.0% |
| Antipsychotic | 1 | -0.39 | -0.19, 0.96 | 0.1854 | NA |
| Novel Class | 0 | -NA | NA | NA | NA |
| SSRI | 8 | -1.30 | -1.13, 1.46 | 0.0000 | 70.1% |
| Tricyclic | 14 | -0.56 | -0.38, 0.74 | 0.0000 | 0.0% |
| Other | 7 | -0.18 | -0.03, 0.40 | 0.1017 | 23.1% |
| **Treatment Duration** (weeks) | 40 | -0.04 | -0.01, 0.06 | 0.0033 | 13.6% |
| **Concomitant Medication** |  |  |  |  |  |
| Yes | 7 | 0.20 | -0.06, 0.45 | 0.1250 | 0.0% |
| No | 17 | 0.21 | -0.06, 0.37 | 0.0062 | 0.0% |
| Not Reported | 16 | 1.03 | -0.86, 1.19 | 0.0000 | 49.2% |
|  |  |  |  |  |  |
| **Study Characteristics** |  |  |  |  |  |
| **Adherence** | 37 | 1.12 | -0.06, 2.31 | 0.0622 | 6.5% |
| **Time Period** |  |  |  |  |  |
| During Intervention | 18 | -0.63 | -0.40, 0.85 | 0.0000 | 40.6% |
| Post Intervention | 21 | -0.40 | -0.18, 0.62 | 0.0003 | 0.0% |
| Follow Up | 1 | -0.65 | -0.41, 1.72 | 0.2304 | NA |
| **Depression Measure** |  |  |  |  |  |
| HAM-D | 17 | -0.44 | -0.19, 0.70 | 0.0006 | 0.0% |
| BDI | 2 | -0.51 | -0.24, 1.27 | 0.1855 | NA |
| MADRS | 18 | -0.59 | -0.37, 0.81 | 0.0000 | 47.8% |
| RDRS | 2 | 0.38 | -0.45, 1.21 | 0.3694 | NA |
| PHQ-9 | 1 | -0.28 | -0.67, 1.23 | 0.5601 | NA |
